# Plan comparison of prostate stereotactic radiotherapy in spacer implant patients

**DOI:** 10.1002/acm2.13387

**Published:** 2021-08-06

**Authors:** Yuya Oki, Kazuyuki Uehara, Kazufusa Mizonobe, Hiroaki Akasaka, Yuichirou Shiota, Risako Sakamoto, Aya Harada, Keiji Kitatani, Tomonori Yabuuchi, Shuichirou Miyazaki, Takayuki Hattori, Hiroshi Mayahara

**Affiliations:** ^1^ Division of Radiation Oncology Kobe Minimally Invasive Cancer Center Kobe Hyogo Japan; ^2^ Division of Radiation Oncology Kobe University Graduate School of Medicine Kobe Hyogo Japan; ^3^ Division of Radiological Technology Kobe Minimally Invasive Cancer Center Kobe Hyogo Japan

**Keywords:** hydrogel spacer, plan comparison, prostate cancer, SBRT

## Abstract

In prostate stereotactic body radiation therapy (SBRT), hydrogel spacers are increasingly used. This study aimed to perform a dosimetry comparison of treatment plans using CyberKnife (CK), commonly used for prostate SBRT, Helical TomoTherapy (HT), and TrueBeam (TB) in patients with hydrogel spacer implantations. The data of 20 patients who received hydrogel spacer implantation for prostate SBRT were retrospectively analyzed. The prescription dose was 36.25 Gy in five fractions to 95% of the planning target volume (PTV; D95). The conformity index (CI), gradient index (GI), homogeneity index (HI), and dose‐volume histogram (DVH) were analyzed for the three modalities, using the same PTV margins. The monitor unit (MU) and the beam‐on‐time (BOT) values were subsequently compared. The CI of TB (0.93 ± 0.02) was significantly superior to those of CK (0.82 ± 0.03, *p* < 0.01) and HT (0.86 ± 0.03, *p* < 0.01). Similarly, the GI value of TB (3.59 ± 0.12) was significantly better than those of CK (4.31 ± 0.43, *p* < 0.01) and HT (4.52 ± 0.24, *p* < 0.01). The median doses to the bladder did not differ between the CK and TB (V18.1 Gy: 16.5% ± 4.5% vs. 15.8% ± 4.4%, *p* = 1.00), but were significantly higher for HT (V18.1 Gy: 33.2% ± 7.3%, *p* < 0.01 vs. CK, *p* < 0.01 vs. TB). The median rectal dose was significantly lower for TB (V18.1 Gy: 5.6% ± 4.5%) than for CK (V18.1 Gy: 11.2% ± 6.7%, *p* < 0.01) and HT (20.2% ± 8.3%, *p* < 0.01). TB had the shortest BOT (2.6 min; CK: 17.4 min, HT: 6.9 min). TB could create treatment plans dosimetrically comparable to those of CK when using the same margins, in patients with hydrogel spacers.

## 1 INTRODUCTION

Prostate cancer is one of the most common cancers in men, and various radiotherapy techniques and regimens are used to treat localized prostate cancer.^1^, ^2^, ^3^, ^4^, ^5^ Recently, the hypo‐fractionated (20–28 fr) intensity‐modulated radiotherapy (IMRT) technique has been increasingly used.^2^, ^3^ Moreover, the usefulness of stereotactic body radiation therapy (SBRT), an ultra‐hypo‐fractionated technique (4–5 fr), has been reported.^4^, ^5^ As the *α*/*β* ratio of prostate cancer is presumed to be low (1.5 Gy), hypofractionation regimens are biologically favorable because of the potentially greater sensitivity of the high radiation dose per fraction.^6^, ^7^Compared with the conventional technique, a steeper dose falloff is achieved with the SBRT technique, which minimizes the radiation dose to the nearby normal tissues. In prostate cancer, intrafractional organ motion should be considered to ensure optimal target coverage.^8^ When SBRT is adopted, it is also crucial to spare the surrounding organs at risk (OARs) as much as possible because of using a high dose per fraction. In the treatment of prostate cancer, rectal sparing is especially important. For prostate SBRT, CyberKnife (CK; Accuray Inc.) is often used because of its ability to track the prostate during irradiation, which allows a highly conformal dose distribution.^9^, ^10^, ^11^, ^12^, ^13^ However, CK is not available at all radiotherapy centers.

Nowadays, we can use the SBRT technique more safely because of the advent of rectal hydrogel spacers.^14^, ^15^, ^16^ With the advent of these spacers, there is an increasing interest in prostate SBRT using a conventional linear accelerator (Linac), which cannot use the tumor tracking method of the CK.^17^ Some studies have compared the dose distribution between CK and other techniques in cases without hydrogel spacers.^18^, ^19^ Bijina et al. reported that CK has higher rectal and bladder doses compared with volumetric modulated arc therapy (VMAT).^18^ Scobioala et al. reported that CK is associated with higher rectal and bladder doses than VMAT and helical TomoTherapy (HT; Accuray Inc.).^19^ However, to the best of our knowledge, few reports compare treatment plans for prostate SBRT in patients with hydrogel spacer placement. Thus, we compared treatment plans among three modalities, namely, CK, HT, and VMAT (TrueBeam; TB, Varian Medical Systems), in patients with hydrogel spacers.

## 2 METHODS

### 2.1 Patients

**FIGURE 1:**
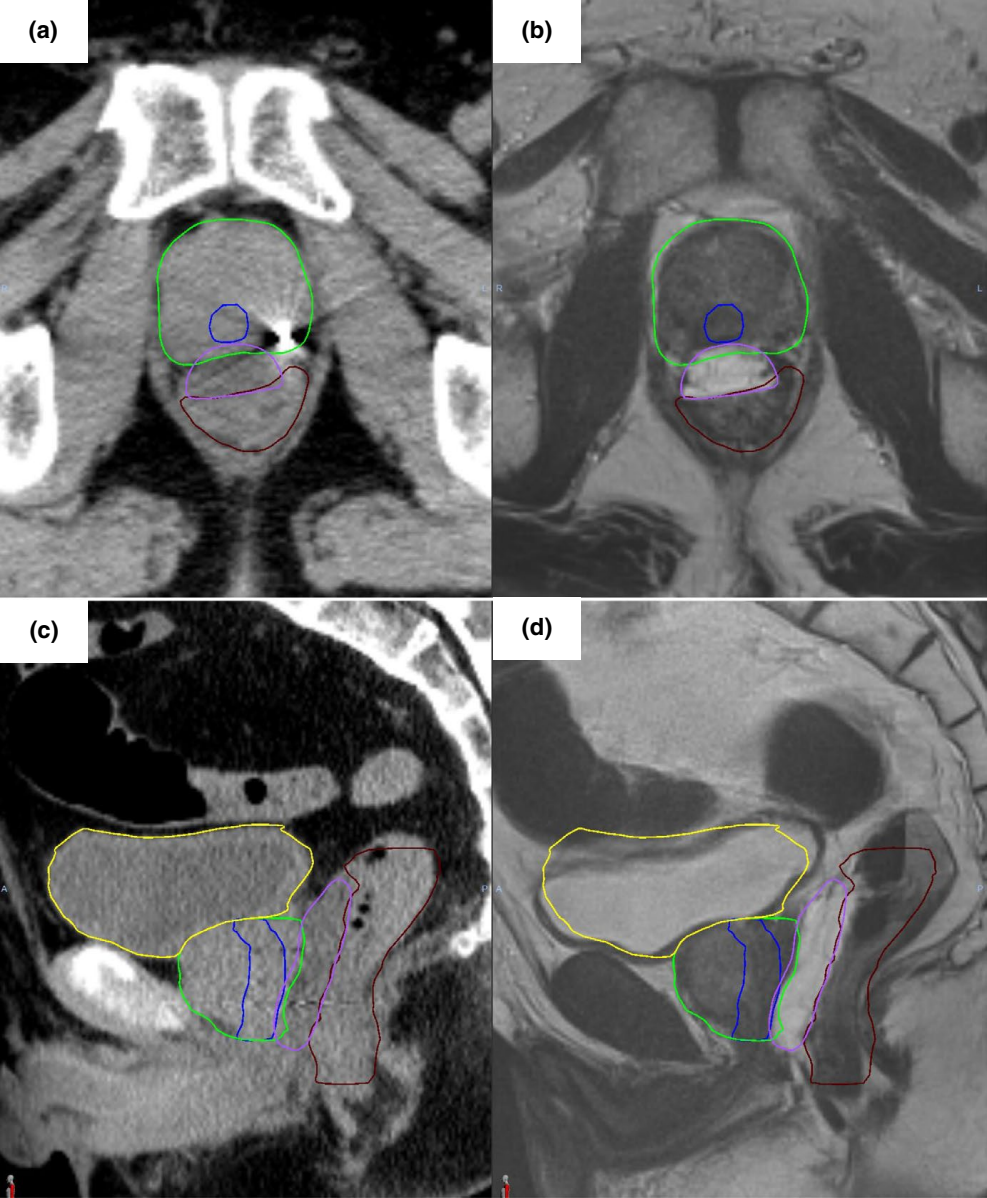
Example of hydrogel spacer implantation. Mid‐gland axial section of the prostate SBRT contours showing the bladder (yellow), urethra (blue), CTV (green), hydrogel spacer (purple), and rectum (brown) on CT simulation scan (a) and fused T2‐weighted MRI images (b). Corresponding sagittal views are also presented (c–d). SBRT, stereotactic body radiotherapy; CTV, clinical target volume; CT, computed tomography; MRI, magnetic resonance imaging

A retrospective study was performed involving 20 patients with localized prostate cancer. Treatment plans were generated for patients treated with CK in our center from August 2020 to March 2021. Informed consent was obtained from the patients for publication of this report and accompanying images. Patient and tumor characteristics are presented in Table 1. Under transrectal ultrasound guidance, all patients underwent transperineal insertion of the hydrogel spacer (SpaceOAR, Boston Scientific). Typical computed tomography (CT) and fused T2‐weighted magnetic resonance imaging (MRI) after spacer implantation are presented in Figure 1. The volume of injected hydrogel spacer was 10 cc, and the median spacer thickness was 12.1 mm (Table 1). Concomitant with the spacer insertion, a urologist transperineally implanted three gold seeds into the prostate gland as internal markers for image guidance.

**TABLE 1:** Variables in the patient population

Characteristics	
No. of patients	20
Median age (range) (years)	73 (54–86)
Median iPSA (range) (ng/mL)	7.96 (4.70–16.98)
T stage	
T1c	5
T2a	8
T2b	3
T2c	2
T3a	2
Gleason score	
3+3 = 6	7
3+4 = 7	5
4+3 = 7	3
4+4 = 8	5
Hormone treatment +/−	14/6
Median CTV volume (range) (cc)	35.6 (17.5–82.1)
Median PTV volume (range) (cc)	58.3 (31.2–119.6)
Median bladder volume (range) (cc)	143.5 (91.9–229.5)
Median rectum volume (range) (cc)	36.6 (24.2–56.9)
Median hydrogel spacer thickness (range) (mm)	12.1 (10.0–15.9)

Abbreviations: CTV, clinical target volume; PSA, prostate‐specific antigen; PTV, planning target volume.

### 2.2 Contouring

CT (Aquilion64, Canon Medical Systems) images were acquired with 1‐mm slice thickness in the supine position using the VacLok system (CIVCO Medical Solutions). Before CT simulation, the patients were required to hold the urine for 1 h or more to have a comfortably full bladder (bladder volume, 100–150 ml). Before urinary collection, they were also asked to empty the rectum. On the day of CT simulation, a T2‐weighted MRI (Vantage Titan, Canon Medical Systems) was acquired and fused with the CT image using Velocity, version 3.2.1 (Varian Medical Systems). In low‐risk disease, the prostate gland alone was delineated as the clinical target volume (CTV). In the intermediate‐ and high‐risk cases, proximal 1 or 2 cm of the seminal vesicles was included in the CTV, depending on the risk classification. To delineate the planning target volume (PTV), according to our institution protocol, a 4‐mm margin in the left/right direction and 3‐mm margins in all the other directions were added. The same PTV margin was used regardless of modality. The rectum, bladder, penile bulb, urethra, testicles, and femoral heads were contoured as organs at risk. The rectum was delineated within 1 cm superior and inferior to the existing PTV plane. The entire structure set was contoured with Velocity and exported to each treatment planning system.

### 2.3 Planning

The prescription dose was 36.25 Gy to 95% of the PTV in five fractions. The maximum dose of the PTV was allowed to be 125% of the prescription dose. The same critical structure dose constraints were applied to the three different techniques. Dose constraints are presented in Table 2.

**TABLE 2:** Dose constraints

Target		
CTV	D99	<36.25 Gy
OAR		
Urethra	D0.03 cc	<40 Gy
	V35 Gy	>95%
Rectum	V36 Gy	<1 cc
	V32.6 Gy	<10%
	V29.0 Gy	<20%
	V27.2 Gy	<25%
	V18.1 Gy	<40%
Bladder	V37 Gy	<5 cc
	V18.1 Gy	<50%

Abbreviations: CTV, clinical target volume; OAR, organ at risk.

CK plans were created using the voxel‐less optimization (VOLO) algorithm on Precision, version 2.0.1.1 (Accuray Inc.). Dose calculations were performed using the ray‐tracing algorithm, and the calculation voxel size was 1 × 1 × 1 mm^3^. The plans were created for CyberKnife VSI, which delivers a 6‐MV photon beam with a dose rate of 1,000 monitor unit (MU)/min. An Iris variable aperture collimator (10–60 mm at 80 cm source to axis distance, SAD) was used. Beam‐on‐time (BOT) was targeted at ˂20 min.

HT plans were generated in Planning Station, version 5.1.1.6 (Accuray Inc.), with a collapsed cone convolution/superposition algorithm. The plan parameters used were 2.5‐cm fixed jaw, pitch of 0.172, modulation factor of 1.7–2.0, and calculation grid of 1.91 × 1.91 × 1 mm^3^. The plans were generated for the TomoHD system, which has a helical 6‐MV photon beam with an 850 MU/min dose rate modulated using 64 binary multi‐leaf collimators.

TB plans were created using the Eclipse Treatment Planning System, version 11.0.31 (Varian Medical Systems). Dose distributions were calculated using a 6‐MV flattening‐filter‐free (FFF) beam and the analytical anisotropic algorithm (AAA). The calculated grid size was 2 × 2 × 1 mm^3^. Each plan consisted of two full coplanar arcs with collimator angle rotations of 30° and 330°. We used the arc geometry tool for the creation of the arcs. The plans were generated for the TrueBeam linear accelerator equipped with a millennium 120 multi‐leaf collimator (MLC) (min leaf width, 0.5 cm). The maximum dose rate was 1400 MU/min.

### 2.4 Plan evaluation

The plan quality was evaluated on Velocity by comparing the dosimetry results obtained from the cumulative dose‐volume histograms (DVH) of the three plans. PTV was evaluated by *D*_98%_, *D*_50%_, and *D*_2%_. The conformity index (CI), dose gradient index (GI), and homogeneity index (HI) were used to compare the dosimetry indices.^20^, ^21^, ^22^ The CI was defined as follows:

$$\text{CI}={\text{TV}}_{\text{PIV}}^{2}/\left(\text{TV}\times V_{\text{RI}}\right),$$

where PIV, V_RI_, and TV correspond to the prescription isodose volume, volume encompassed within the reference isodose, and target volume, respectively.

GI was defined as follows:

$$\text{GI}={\text{PIV}}_{50}/\text{PIV},$$

where PIV_50_ corresponds to the volume receiving at least 50% of the prescription dose.

The HI was defined as follows:

$$\text{HI}=\left(D_{2\%}‐D_{98\%}\right)/D_{50\%}.$$

Moreover, the average number of MUs and the BOT were compared among the three techniques.

### 2.5 Statistical analysis

Data are presented as mean ± standard deviation. All statistical analyses were performed using R, version 4.0.2 (R Foundation for Statistical Computing).^23^ Differences between the groups were analyzed using the Friedman test followed by pairwise post hoc comparisons using the Wilcoxon signed rank test with Bonferroni correction. Data were considered statistically significant at *p* < 0.05.

## 3 RESULTS

### 3.1 DVH parameters and dose distribution

**FIGURE 2:**
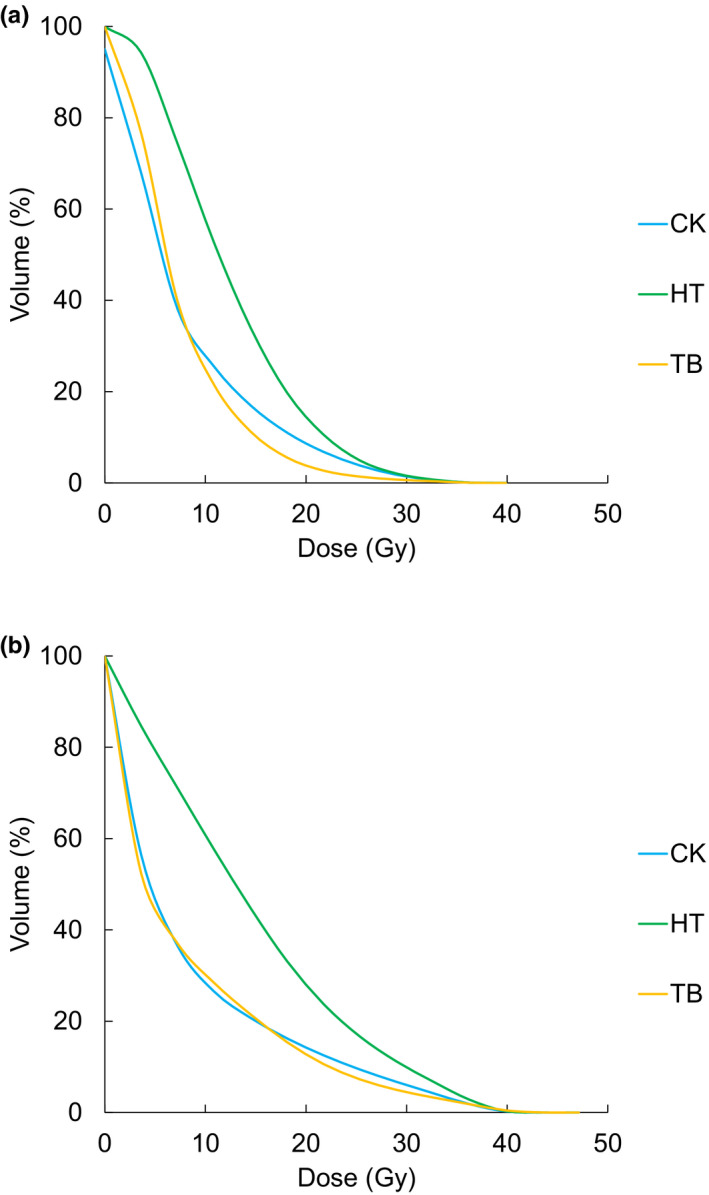
Comparison of the rectum and bladder using a DVH. The average DVH of the rectum (a) and the bladder (b). DVH, dose–volume histogram

**FIGURE 3:**
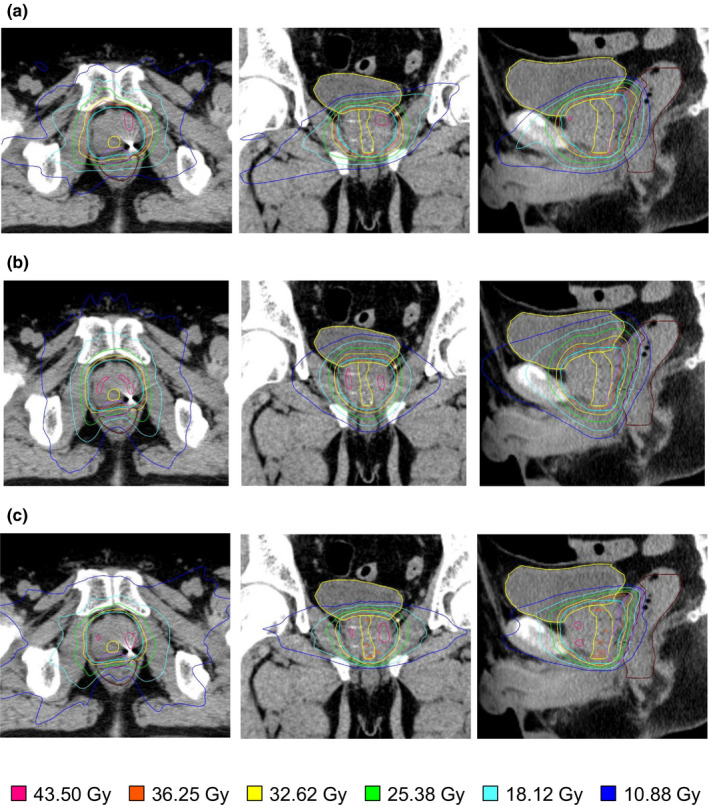
Typical dose distribution for a patient with CK, HT, and TB. Typical dose distribution planned for one patient with CyberKnife (a), Helical TomoTherapy (b), and TrueBeam (c). The contours show the PTV (light blue), bladder (yellow), rectum (brown), hydrogel spacer (purple), and urethra (yellow). CK, CyberKnife; HT, Helical TomoTherapy; TB, TrueBeam; PTV, planning target volume

Table 3 shows the DVH parameters for all treatment modalities. Regarding PTV and CTV dose indices, there was a significant difference between some indices. Concerning the dose to the OARs, DVH values in the rectum and bladder are shown in Figure 2. Concerning the high‐dose (V36.0 Gy, V32.6 Gy) volumes to the rectum, there was no significant difference among the modalities. The average V18.1 Gy of the rectum was 11.2 ± 6.7%, 20.2 ± 8.3%, and 5.6 ± 4.5% for CK, HT, and TB, respectively. TB showed significantly superior rectal sparing compared with CK (*p* < 0.01) and HT (*p* < 0.01). There was no significant difference between CK and TB regarding the average bladder V18.1 Gy. Conversely, it was significantly higher in HT (*p* < 0.01 vs. CK, *p* < 0.01 vs. TB). The dose distribution in the three techniques for one representative patient is presented in Figure 3. TB demonstrated the steepest dose fall off for the rectal side. For HT, compared with the other modalities, a gentle dose gradient was observed in the craniocaudal direction of the PTV.

**TABLE 3:** DVH parameters among the different treatment modalities

		CK	HT	TB	(p‐value)		
		Mean ± SD (range)	Mean ± SD (range)	Mean ± SD (range)	CK vs. HT	HT vs. TB	TB vs. CK
PTV	D_98%_ (Gy)	35.1 ± 0.2 (34.7–35.5)	35.3 ± 0.3 (34.7–35.8)	35.5 ± 0.1 (35.2–35.7)	0.20	0.02	<0.01
	*D*_50%_ (Gy)	39.7 ± 0.4 (39.1–40.5)	39.6 ± 0.5 (38.9–40.4)	39.9 ± 0.2 (39.5–40.3)	0.70	0.05	0.05
	*D*_2%_ (Gy)	44.4 ± 0.7 (42.9–45.4)	43.8 ± 0.4 (43.2–44.5)	43.6 ± 0.4 (42.9–44.2)	<0.01	0.30	<0.01
CTV	*D*_99%_ (Gy)	36.8 ± 0.3 (36.3–37.3)	36.6 ± 0.5 (36.0–38.2)	36.4 ± 0.3 (36.1–37.0)	0.63	0.32	<0.01
Urethra	D0.03 cc (Gy)	38.8 ± 0.5 (37.7–39.8)	38.0 ± 0.6 (37.3–39.2)	38.2 ± 0.6 (37.1–39.0)	<0.01	0.75	<0.01
	V35.0 Gy (%)	99.6 ± 0.7 (97.5–100)	100.0 ± 0.0 (100)	100.0 ± 0.1 (99.5–100.0)	0.02	1.00	0.07
Rectum	V36.0 Gy (cc)	0.0 ± 0.1 (0–0.5)	0.0 ± 0.1 (0–0.5)	0.0 ± 0.1 (0–0.4)	1.00	1.00	1.00
	V32.6 Gy (%)	0.7 ± 1.4 (0–6.2)	0.7 ± 2.0 (0–8.7)	0.4 ± 1.1 (0–4.7)	1.00	1.00	1.00
	V29.0 Gy (%)	1.8 ± 2.6 (0–10.5)	2.1 ± 3.8 (0–16.0)	0.8 ± 1.8 (0–7.5)	1.00	<0.01	<0.01
	V27.2 Gy (%)	2.7 ± 3.3 (0–12.5)	3.2 ± 4.8 (0.1–19.6)	1.1 ± 2.1 (0–8.9)	1.00	<0.01	<0.01
	V18.1 Gy (%)	11.2 ± 6.7 (0.7–24.6)	20.2 ± 8.3 (8.6–40.0)	5.6 ± 4.5 (0–20.0)	<0.01	<0.01	<0.01
	V7.25 Gy (%)	37.9 ± 10.8 (12.3–56.3)	74.4 ± 10.7 (56.0–87.8)	39.1 ± 10.7 (23.7–62.8)	<0.01	<0.01	1.00
Bladder	V37 Gy (cc)	1.9 ± 0.9 (0.5–3.8)	2.8 ± 1.7 (0.7–6.5)	2.1 ± 1.0 (0.6–4.5)	<0.01	0.05	0.06
	V18.1 Gy (%)	16.5 ± 4.5 (8.5–24.8)	33.2 ± 7.3 (22.9–51.1)	15.8 ± 4.4 (8.0–23.7)	<0.01	<0.01	1.00

Abbreviations: CK, CyberKnife; CTV, clinical target volume; DVH, dose–volume histogram; HT, helical TomoTherapy; PTV, planning target volume; TB, TrueBeam.

### 3.2 Dosimetric indices

Table 4 shows the dosimetry parameters for the three modalities. A paired comparison revealed a significantly better CI for TB (0.93 ± 0.02) than for CK (0.82 ± 0.03, *p* < 0.01) and HT (0.86 ± 0.03, *p* < 0.01). HT demonstrated a significantly superior CI than CK (*p* < 0.01). Similarly, TB showed a significantly superior GI (3.59 ± 0.12) than CK (4.31 ± 0.43, *p* < 0.01) and HT (4.52 ± 0.24, *p* < 0.01). There was no significant difference between CK and HT (*p* = 0.32) for the GI.

**TABLE 4:** Dosimetry and delivery efficiency parameters among the different treatment modalities

	CK	HT	TB	(*p*‐value)		
	Mea ± SD (range)	Mean ± SD (range)	Mean ± SD (range)	CK vs HT	HT vs TB	TB vs CK
CI	0.82 ± 0.03	0.86 ± 0.03	0.93 ± 0.02	<0.01	<0.01	<0.01
	(0.76–0.88)	(0.80–0.90)	(0.90–0.98)			
GI	4.31 ± 0.43	4.52 ± 0.24	3.59 ± 0.12	0.32	<0.01	<0.01
	(3.59–5.03)	(4.18–4.94)	(3.39–3.84)			
HI	0.23 ± 0.02	0.22 ± 0.01	0.20 ± 0.01	0.01	<0.01	<0.01
	(0.19–0.27)	(0.20–0.24)	(0.18–0.23)			
MU	4982.9 ± 785.0	5847.1 ± 526.9	3637.8 ± 460.6	<0.01	<0.01	<0.01
	(3210.4–6278.4)	(4896.0–7013.0)	(2568.9–4200.0)			
BOT (min)	17.4 ± 2.0	6.9 ± 0.6	2.6 ± 0.3	<0.01	<0.01	<0.01
	(17.0–21.0)	(5.8–8.2)	(1.8–3.0)			

Abbreviations: BOT, beam‐on‐time; CI, conformity index; CK, CyberKnife; GI, dose gradient index; HI, homogeneity index; HT, helical TomoTherapy; MU, monitor unit; TB, TrueBeam.

### 3.3 Delivery efficiency

Table 4 shows the number of MUs and BOTs of each technique. While CK showed the largest BOT value among the three techniques (17.4 ± 2.0 min), TB had the fastest BOT among the three modalities (2.6 ± 0.3 min).

## 4 DISCUSSION

For all modalities, it was possible to create clinically acceptable plans that met the dose constraints for most patients. In prostate SBRT, CK is generally employed because it can use a real‐time tracking method.^9^, ^10^, ^11^, ^12^ However, as aforementioned, some studies have shown few distinct dosimetry advantages in choosing CK over VMAT in patients without hydrogel spacer implantation.^18^, ^19^ Similarly, in cases of hydrogel spacer implantation, our results showed that TB‐VMAT could create treatment plans dosimetrically equivalent to those of CK when the same treatment margins were used.

When CK is used, it generally takes 35–50 min to deliver prostate SBRT, including a 5‐min setup time.^24^, ^25^ However, in our study, the average BOT was only 17 min for CK. The VOLO optimizer, a new optimization algorithm for CK, can reduce the BOT more than the previous Sequential optimization algorithm.^26^ Moreover, in hydrogel spacer implantation, it becomes easy to reduce the rectal dose to a clinically acceptable level. Saito et al. compared the CK plans with and without hydrogel spacer. Interestingly, they reported that the D_2%_ values of the rectum were 36.10 ± 1.52 and 24.33 ± 1.81 Gy without and with the spacer, respectively, when using more than 150 beams.^16^ In our study, the average number of beams was 77 (range, 51–96). Although we used a small number of beams, the rectal dose constraints were easily achieved. As the number of beams is reduced, the MU per beam increases, which leads to an expansion of the middle dose region and worsening of the GI. In CK, various plans can be created depending on the optimization parameters.^27^ When we increase the number of beams allowing for longer BOT, the rectal dose would be further reduced. Recently, CK has been equipped with InCise MLC. Kathriarachchi et al. reported that the device could provide dosimetrically equivalent plans using less BOT.^25^

Like CK, we could create HT plans with shorter BOT and smaller MUs than previously reported. Bijina et al. reported that the average BOT of HT was 11.1 min.^18^ However, in our study, this value was only 6.9 min. The shorter treatment time resulted in lower intra‐fractional motion of the prostate.^8^, ^28^, ^29^ If the spacer is inserted, we can create clinically acceptable treatment plans in a shorter BOT. Thus, the hydrogel spacer effectively reduces treatment uncertainty when treating with HT, which cannot use a tumor tracking method. However, the bladder dose was significantly higher in HT than in the other modalities. Our TomoHD is not equipped with TomoEdge, which can reduce the longitudinal penumbra by varying the width of the jaw in the superior and inferior directions.^30^ Therefore, if TomoEDGE technology was used, it could have improved the bladder dose. The fixed jaw also worsens GI because of the median dose spread in the superior and inferior directions.

In this study, we could create a better dose distribution than the other two modalities by using TB. However, it is not necessarily the best modality for prostate SBRT in clinical situations. For prostate cancer external‐beam radiation therapy, an intrafractional prostate motion must be considered.^8^ This is particularly true when using the SBRT technique, where organ motion has a greater impact on irradiation accuracy because of the high dose delivery per fraction. Thus, the real‐time tracking system of CK could be advantageous for treatment accuracy.^31^ During VMAT treatments, irradiation accuracy can be improved by performing a positional correction using a kV imaging device (ex. OBI) before irradiation of each field or by monitoring the prostate motion using an electromagnetic localization device (ex. Calypso).^32^, ^33^ Prostate SBRT with VMAT often uses PTV margins 3‐mm in the posterior direction and 5‐mm in all the other directions.^5^, ^34^ Tree et al. showed that in patients without hydrogel spacer implantation, rectal constraints failed in some patients when VMAT plans were created with larger margins compared to those of CK.^35^ In our study, we used the same margins regardless of the modalities and image guidance methods to eliminate the effect of different margins. With hydrogel spacers, it is possible to make a space of 12.1 mm between the rectum and the CTV. Therefore, the rectal dose constraint will not fail even when using a larger PTV margin. The hydrogel spacer may make it feasible to perform prostate SBRT more safely and less uncertainly than without spacer when using universal Linac, such as TB and HT.

This study had some limitations. First, all treatment plans were created by a single dosimetrist. Future work will be needed to compare treatment plans with different margins depending on the tracking methods and treatment time. Furthermore, we only investigated cases that used the hydrogel spacer. Therefore, it is necessary to make comparisons between the three modalities with and without using spacers.

## 5 CONCLUSION

In this study, by using CK and HT, prostate SBRT could be performed in a shorter treatment time and with lower rectal doses in patients with hydrogel spacers, compared with the corresponding reported by previous studies on patients without spacers. TB can create treatment plans dosimetrically comparable to those of CK when using the same margins.

## CONFLICT OF INTEREST

The authors declare that there are no conflicts of interest.

## AUTHOR CONTRIBUTIONS

YO, KU, KM, HA, and HM were involved in study design and data interpretation. YO, YS, RS, AH, KK, TY, SM, and TH were involved in data acquisition and analysis. All authors critically revised the report, commented on drafts of the manuscript, and approved the final report.

## Data Availability

Research data are stored in an institutional repository and will be shared upon request to the corresponding author.
